# The INventory of Callous-Unemotional Traits and Antisocial Behavior (INCA) for Young People: Development and Validation in a Community Sample

**DOI:** 10.3389/fpsyg.2019.00713

**Published:** 2019-03-27

**Authors:** Fabia Morales-Vives, Sandra Cosi, Urbano Lorenzo-Seva, Andreu Vigil-Colet

**Affiliations:** Research Center for Behavior Assessment, Department of Psychology, Universitat Rovira i Virgili, Tarragona, Spain

**Keywords:** psychopathy, callous-unemotional traits, antisocial behavior, adolescence, response biases

## Abstract

Callous-unemotional traits are defined as potential markers of psychopathy in children and adolescents. Previous studies with the most widely used instrument designed specifically to assess these traits, the Inventory of Callous-Unemotional Traits (ICU), have shown major methodological problems. For this reason, the purpose of the present study was to develop a valid and reliable test to assess callous-unemotional traits for the adolescent population free of the response biases social desirability (SD) and acquiescence (AC). In order to obtain responses free of these biases, we used SD item markers as well as content balanced items to identify a factor related to SD and AC, so that SD and AC effects can be removed from the individual scores on content factors. As well as the CU traits (unemotional, callousness, and uncaring scales), this new questionnaire also contains an additional scale for assessing antisocial behaviors. The test was administered to 719 adolescents between 13 and 19 years old. Exploratory and confirmatory factor analysis yielded the following expected four dimensions with a good fit: Unemotional, Callousness, Uncaring, and Antisocial Behavior. These scales also showed good psychometric properties with good reliability, and convergent, discriminant and criterion validity.

## Introduction

Psychopathic behavior is a complex phenomenon that, together with aggressive behavior, raises noticeable concerns in society and is exacerbated by the spread of violent images in the media. For this reason, a considerable amount of research has been carried out to understand and describe psychopathy. However, there is still some debate about some issues related to psychopathy – for example, the relevance of genetic and environmental influences (e.g., Viding et al., 2005; Henry et al., 2016), the possible role of criminal behavior as a central component of psychopathy (Skeem and Cooke, 2010), or the structure of psychopathy (Blackburn, 2007) – but there is basic agreement on its most important emotional and behavioral characteristics (Hare et al., 2000). More specifically, in the area of interpersonal relationships, psychopaths can be defined as insensitive, arrogant, domineering, and manipulative; on the emotional level they show a lack of remorse and empathy, which makes it difficult for them to establish strong emotional relationships, and their lifestyle is characterized by impulsive and irresponsible behavior and a tendency to ignore and violate social norms (Abalos et al., 2004).

Psychopathy manifests at a relatively early age and it tends to be relatively stable throughout life (Lynam, 1996; Frick et al., 2003b). In fact, there is evidence that the origins of aggressive behavior can be traced back to preschool years (Loeber and Farrington, 2000), and psychopathic traits can emerge in children as young as 4 years old (Dadds et al., 2005). However, its biological basis and the environmental factors involved in its development and which favor its relative stability throughout life are still not well understood (Frick et al., 2003b).

Psychopathy in children and adolescents has traditionally been studied using the approach of callous-unemotional (CU) traits. These traits are the potential markers that might be the precursors to the development of psychopathy: lack of empathy, lack of guilt and regret, the manipulation of others, irresponsible attitude regarding self-performance, and poverty of emotional expression (Frick, 2004). Children with high levels of CU traits are deficient in emotional empathy but not in cognitive empathy, as they seem to understand the perspective of the others but they do not emotionally resonate with their feelings (Waller et al., 2015; Waller and Hyde, 2018). Moreover, many studies have found that CU traits are related to a range of dysfunctional behaviors in adolescents. For instance, young people with higher scores in CU tend to show greater antisocial behavior and repeated behavior problems (Frick et al., 2003a, 2005). In this regard, the latest version of the *Diagnostic and Statistical Manual of Mental Disorders* (DSM-5; American Psychiatric Association, 2013) includes CU traits as severity markers in the diagnostic criteria for Conduct Disorder. Youngsters with CU traits also show higher levels of criminal behavior involving aggression and sexual violence (Caputo et al., 1999; Frick and White, 2008), and higher levels of premeditated, instrumental violence (Frick et al., 2003a; Pardini et al., 2003). Moreover, higher levels of delinquency related to substance abuse are observed in these young people (Taylor and Lang, 2006). The origin of these antisocial behaviors shown by adolescents with CU traits may be related to deficits in processing negative emotional stimuli (Blair, 1999; Blair et al., 2001; Kimonis et al., 2006). In fact, they are less inhibited by fear and anxiety (Frick et al., 1999; Lynam et al., 2005) and less affected by threat of punishment (Fisher and Blair, 1998; Barry et al., 2000). All these studies show the relevance of CU traits and how important it is that they are assessed reliably.

In 2004, Paul J. Frick developed the 24-item Inventory of Callous-Unemotional Traits (ICU), on the basis of the four items of the Antisocial Process Screening Device (APSD; Frick and Hare, 2001) which loaded consistently on the CU scale. Although there was another instrument with a CU scale, the Youth Psychopathic trait Inventory (YPI; Andershed et al., 2002), the ICU is the only questionnaire that specifically assesses CU traits.

Despite the significance of CU traits and the importance of assessing them, many methodological issues affect the development of this test. As far as the factor structure is concerned, it should be taken into account that Frick (2004) developed the ICU to assess four different facets of psychopathy: Careless, Uncaring, Unemotional, and Callous behaviors. However, he did not provide any evidence about the dimensionality of the instrument. Several authors have carried out studies on the factor structure of the ICU, most of which have found a three-factor structure consisting of Unemotional, Callousness, and Uncaring traits (Essau et al., 2006; Kimonis et al., 2008, 2013; Fanti et al., 2009; Roose et al., 2010; Ciucci et al., 2014; López-Romero et al., 2015), but this factor structure presents several problems. In fact, the exploratory factor analyses were done using Pearson correlation coefficients instead of polychoric correlations, which are more advisable with Likert-type items (Muthen and Kaplan, 1985), and most of them used procedures such as the scree test to determine the number of factors to retain instead of more appropriate methods such as parallel analysis. In some cases, however, (for example, the study by Feilhauer et al., 2012), principal component analysis was used instead of factor analysis. The confirmatory analyses carried out by various studies show no clear consensus, and many of them reached a marginal fit only after applying such highly undesirable procedures as allowing a high number of error terms to correlate after the modification indexes had been inspected (Essau et al., 2006; Houghton et al., 2013; Ciucci et al., 2014), or some items (Kimonis et al., 2008; López-Romero et al., 2015) or even scales have been removed (Feilhauer et al., 2012; Houghton et al., 2013). These “*ad hoc*” procedures often lack substantive and theoretical foundation, and are likely to capitalize on chance (Ferrando and Lorenzo-Seva, 2000).

There are also some doubts about the content of the scales found by the factor analyses of most studies (e.g., Essau et al., 2006; Fanti et al., 2009; Ciucci et al., 2014; López-Romero et al., 2015), because some of the items in a specific scale seem to be related to a different scale. For instance, the Uncaring scale contains some items that seem to be more related to callousness, because they refer to a lack of empathy or remorse (i.e., I feel bad or guilty when I do something wrong, or I try not to hurt others’ feelings). In fact, some items in the Callousness scale also refer to a lack of empathy or remorse, like those other items in the Uncaring scale (i.e., I do not feel remorseful when I do something wrong, or I am concerned about the feelings of others). Likewise, the Callousness scale also contains some items that seem to be more related to uncaring, because they refer to a lack of responsibility (i.e., I do not care about doing things well, or I do not care about being on time). Also, some items in the Uncaring scale refer to responsibility (i.e., I work hard on everything I do, or I care about how well I do at school or work).

Other studies have proposed a different factorial structure. For instance, Houghton et al. (2013) proposed that fit was best using a two-factor model and removing the items of the Unemotional scale, while others, such as Feilhauer et al. (2012), failed to confirm the previously reported factorial structures and proposed a new exploratory analysis, which yielded a five-factor structure. Taking all this into account, these factorial structure problems seem to indicate that the three-factor structure proposed initially by Essau et al. (2006) may not be suitable, which may also explain why in many cases researchers have used only the ICU’s overall score instead of the scores of specific scales (e.g., Stickle et al., 2009; Viding et al., 2009; White et al., 2009).

We also have to take into account that some characteristic features of psychopathy, such as manipulating others for one’s own ends, are considered to be socially undesirable. For this reason, like other personality traits such as aggressiveness, the items that assess this construct are expected to be deeply impacted by social desirability (SD) (Rogers et al., 2002; Vigil-Colet et al., 2012). Several studies show that controlling acquiescence (AC) in personality questionnaires provides a simpler and more congruent factor structure (Rammstedt and Farmer, 2013; Navarro-González et al., 2016; Morales-Vives et al., 2017). Therefore, response bias control is highly recommended for assessing these kinds of construct, although the ICU test does not control any response bias.

Since CU traits play a major role in the prediction of a wide range of antisocial and problematic behaviors in adolescents and given the scarcity of the measures available and their problems, the main purpose of the present study is to develop a new CU traits questionnaire for the adolescent population free of the response biases SD and AC. As well as the CU traits (unemotional, callousness, and uncaring scales), which are also included in the questionnaire ICU, the new questionnaire also contains an additional scale for assessing antisocial behaviors (challenge to authority and breaking social rules). This scale was included because, according to Hare et al. (2000), one of the key aspects that defines the psychopath is an unstable and antisocial lifestyle. Moreover, including this scale means that the same instrument has a measure of the precursors of psychopathy as well as how they lead to dysfunctional and detrimental behaviors in society, which can be very useful in fields such as forensic, education, etc.

## Materials and Methods

### Development of the INventory of Callous-Unemotional Traits and Antisocial Behavior (INCA)

We wanted the INCA to assess four correlated traits: Unemotional (UE), Callousness (CA), Uncaring (UC), and Antisocial Behavior (AB). Unemotional refers to deficient emotional affect, which implies a lack of emotional expression. Callousness is defined as the lack of empathy and sensitivity to the needs and suffering of others, and involves a tendency to manipulate others for one’s own benefit, feeling little guilt and remorse when others are harmed. Uncaring refers to a lack of responsibility and effort, with a tendency to be lax about one’s own obligations and duties (for example not respecting deadlines or not finishing tasks). Antisocial Behavior refers to the violation of norms and social rules, challenging authority, engaging in behaviors which are illegal or breaking social rules which may harm others or the community.

We wrote an initial pool of 72 items, which was assessed by external judges with experience in personality test development and adolescents. These judges were asked to indicate, for each item, if the content was suitable for assessing the corresponding dimension, if the statement was clear, if the length of the item was adequate and if the response format was compatible with the item statement. The items considered by the external judges to be the most appropriate were administered to 244 adolescents in a pilot study to determine whether they were clear and easy to understand. In this pilot study, those items with loadings lower than λ = 0.30 or with complex loadings (greater than λ = 0.30 on more than one content factor) were removed. The resulting version had 38 content items, plus four markers of SD and a dummy item that we included as the first item of the test so that it could be used as a training item in computer administrations (Ferrando and Lorenzo-Seva, 2005). Therefore, the final version of the test includes a total of 43 items. Item responses are made using a 5-point Likert-type scale ranging from 1 (disagree strongly) to 5 (agree strongly). Moreover, the test is content balanced (half of the items measured in one direction of the trait whereas others measured in the opposite direction). The use of SD markers and reversed items allowed us to implement the procedure developed by Ferrando et al. (2009) and Lorenzo-Seva and Ferrando (2009) to control for potential response biases of SD and AC, which has been successfully implemented in several questionnaires of personality and psychological maturity (Morales-Vives et al., 2013; Vigil-Colet et al., 2013; Ruiz-Pamies et al., 2014). This procedure consists of using SD item markers as well as content balanced items to identify a factor related to SD and AC, so that SD and AC effects can be removed from the individual scores on content factors. More specifically, this procedure has three main steps. The first one consists of identifying a factor related to SD, using the set of markers of SD. The inter-marker correlation matrix is analyzed using factor analysis to obtain the corresponding loading values of each marker in the SD factor. These loading values are then used to compute the loading values of the content items in the SD factor using the Instrumental Variables Technique (Hägglund, 1982), and the variance explained by the SD factor is removed from the inter-item correlation matrix. The second step consists on identifying a factor related to AC. The residual inter-item correlation matrix obtained from the previous step is analyzed to remove the variance due to acquiescent responding from the content items using a totally or partially balanced scale (Lorenzo-Seva and Ferrando, 2009). Finally, the resultant residual inter-item correlation matrix is analyzed using factor analysis to identify the content variables of interest.

### Participants

The participants were 719 adolescents (318 males and 401 females) aged between 13 and 19 years old (*M* = 15.22; *SD* = 1.62) from four high schools in the province of Tarragona (Spain).

### Measures

In addition to the INCA, the following questionnaires were used to assess convergent and discriminant validity:

#### The Inventory of Callous Unemotional Traits (ICU; Frick, 2004)

The Inventory of Callous Unemotional traits (ICU; Frick, 2004), a questionnaire specifically designed to evaluate precursors of psychopathy in youth populations through three dimensions: Callousness (CA), Uncaring (UC), and Unemotional (UE) traits. We used the self-report Spanish adaptation of the ICU developed by López-Romero et al. (2015), which consists of 24 items with a 4-point response format (0 = never/almost never; 3 = always/almost always) with internal consistencies of α = 0.76, α = 0.82, and α = 0.78 for CA, UC, and UE, respectively.

#### The Barratt Impulsiveness Scale-11 for Children (BIS-11c; Chahin et al., 2010)

The BIS-11c consists of 30 items with a 4-point response format (0 = never/almost never; 3 = always/almost always). The questionnaire measures motor (MI), non-planning (N-PI), and cognitive (CI) impulsivity and shows internal consistencies of α = 0.80, α = 0.73, and α = 0.68, respectively.

#### The Overall Personality Assessment Scale (OPERAS; Vigil-Colet et al., 2013)

This 40-item test provides SD- and AC-free scores with good reliability and temporal stability for the factors Extraversion (EX; ρ_θθ_ = 0.86; *r_tt_* = 0.70), Emotional Stability (ES; ρ_θθ_= 0.86; *r_tt_* = 0.70), Conscientiousness (CO; ρ_θθ_= 0.77; *r*_tt_ = 0.75), Agreeableness (AG; ρ_θθ_= 0.71; *r_tt_* = 0.73), and Openness to experience (OE; ρ_θθ_= 0.81; *r_tt_* = 0.79). Temporal stability was calculated from the correlations between the scores obtained by a sample at two different moments in time, 4 weeks apart.

### Procedure

This study was carried out in accordance with the recommendations of the Spanish organic law 15/1999 and the Spanish Agency for Data Protection, which regulate the fundamental right to the protection of data. This project and the protocol was approved by the Ethical Committee of the Faculty of Educational Sciences and Psychology of the Universitat Rovira i Virgili. Moreover, we obtained the parental written informed consent from all subjects. All parents gave written informed consent in accordance with the Declaration of Helsinki.

The questionnaires were administered collectively during regular school hours, in groups of 15–30 students, by professional psychologists. Students were guaranteed anonymity and confidentiality, and participation was voluntary. School approval and parental written informed consent were obtained before the study.

### Data Analysis

The sample of 719 participants was split into two equally representative subsamples of the same population (i.e., all possible sources of variance are enclosed in both subsamples) using the DUPLEX algorithm (Snee, 1977). We computed an exploratory factor analysis (EFA) with the first sample and a confirmatory factor analysis (CFA) with the second (Ferrando and Lorenzo-Seva, 2000). As both analyses led to the same conclusions, a final EFA was carried out with the overall sample to obtain the factorial weights required to compute the participants’ factor scores. These factor analyses were carried out using Psychological Test Toolbox (Navarro et al., in press) and LISREL 8.5 (Jorkeskog and Sorbom, 2001). The structural model was performed using LISREL 9 (Jorkeskog and Sorbom, 2001). We also used SPSS 23.0 to compute sex differences and the convergent and discriminant validity of the INCA questionnaire.

## Results

### Exploratory Factor Analysis

The polychoric correlation matrix was computed for the first sample using 42 items (the first item in the test was excluded from the analyses because it is a dummy item). We used polychoric correlations because they are more suitable for Likert-type scales, and because the skewness or kurtosis of some items was greater than one in absolute value. The Kaiser-Meyer-Olkin (KMO; Kaiser, 1974) index value was 0.86, which indicates that the correlation matrix is suitable for factor analysis.

We computed Optimal Implementation of Parallel Analysis (Timmerman and Lorenzo-Seva, 2011) using 500 random datasets. In this implementation of Parallel Analysis, the decision of the optimal number of factors to be extracted is not based on the eigenvalues themselves, but on the proportion of explained common variance related to the eigenvalues. The results suggested that, as expected, the data had four underlying factors.

We applied the procedure proposed by Ferrando et al. (2009) explained above to determine the response bias factors. Four factors were retained using unweighted least squares (ULS) and an oblique rotation, more specifically a Promin rotation (Lorenzo-Seva, 1999), because we expected the factors to be correlated. We obtained the four content factors that we expected: Unemotional, Callousness, Uncaring, and Antisocial Behavior.

To assess the fit of the rotated loading matrix, the congruence index (Tucker, 1951) was computed between the rotated loading matrix and the ideal loading matrix (i.e., a loading matrix where, for each item, a single loading is one and the other loadings are zero). The congruence values ranged between 0.88 and 0.98. As the coefficients were all above the threshold of 0.85, the factor similarity between the rotated loading matrix and the ideal loading matrix was fair (Lorenzo-Seva and Ten Berge, 2006). We also computed the Goodness of Fit Index (GFI) and the Root Mean Square of Residuals (RMSR), obtaining values of 0.95 and 0.054, respectively. As a GFI equal or higher than 0.95 is considered to be good and Kelly’s criterion indicates that the expected mean value of RMSR for an acceptable model is 0.053, we can conclude that both indices show that the model has a good fit.

Finally, in order to evaluate the simplicity of the factor solution, Bentler’s Simplicity index (S; Bentler, 1977) and the Loading Simplicity index (LS; Lorenzo-Seva, 2003) were computed. The values of the S index and the LS index were 0.99 and 0.60, respectively. These values suggest that the factor simplicity is high, which suggests that each item is mainly related to only one dimension.

### Confirmatory Factor Analysis

We performed a CFA to examine the replicability of the factor structure obtained in the first sample. First the variance due to SD and AC was partialized out following the procedure proposed by Ferrando et al. (2009). ULSs estimates were computed from the residual covariance. It was proposed that the model should retain four correlated factors, as the EFA explained above suggested. The ideal pattern matrix proposed was also the one expected, as in the exploratory analysis.

The values obtained for the fit indexes were CFI = 0.99, GFI = 0.97 and RMSEA = 0.033, so the data seemed to show an acceptable fit to the proposed model. Chi-square test was significant, χ^2^(565) = 1433.85, *p* < 0.01. However, this statistic is very sensitive to sample size, so it cannot be relied upon as a basis for acceptance or rejection (Jöreskog and Sörbom, 1988). The chi-square to degrees of freedom ratio was 2.5. Taking into account that values lower than 2 indicate an excellent fit and values lower than 3 indicate an acceptable fit, our result suggests an acceptable fit (Jöreskog and Sörbom, 1988).

### Final Exploratory Factor Analysis

As both EFA and CFA led to similar conclusions, we used the whole sample (*N* = 719) to estimate the factor loadings and the weights to estimate the participants’ factor scores. We repeated the same analysis as in EFA, but with all the participants so that the sample was the largest possible and the estimates of the weights used to compute individual factor scores were the best possible. The KMO value of 0.88 again indicated that the correlation matrix was suitable for the factor analysis. The GFI index was now 0.95 and the value of RMSR was 0.049. The congruence index values ranged between 0.91 and 0.98. Finally, S and LS were 0.99 and 0.66, respectively. The values obtained in the final factor analysis indicated a good fit to the model.

Table 1 shows the loading values after rotation on both the content scales and the control scales (SD and AC). As can be seen, several items loaded more than λ = 0.30 on the AC factor, two items had loadings greater than λ = 0.30 and six had loadings greater than λ = 0.20 on the SD factor, which justifies using methods to control response biases when assessing these traits. In fact, controlling these biases allowed us to obtain the loadings of the items on the four content factors free of SD and AC (see Table 1). Moreover, as can be seen in this table, each item loads on the expected factor and has low loadings on the other factors. Table 2 shows the interfactor correlation matrix. As can be seen, correlation values among content factors ranged from *r* = 0.02 to *r* = 0.25.

**Table 1:** Loading matrix obtained in the final factor analysis, factor reliabilities and congruence with expected factor solution.

	Control scales		Content scales			
		
Item	SD	AC	UE	CA	UC	AB
18. I have occasionally taken something that is not mine	**0.48**	0.00	0.00	0.00	0.00	0.00
(*Alguna vez he cogido algo que no era mío*)						
27. I have occasionally said bad things about other people	**0.72**	0.00	0.00	0.00	0.00	0.00
(*Alguna vez he dicho algo malo de alguien*)						
33. I have occasionally felt jealous of somebody else	**0.59**	0.00	0.00	0.00	0.00	0.00
(*Alguna vez he sentido envidia de alguien*)						
42. Sometimes I like to gossip about others	**0.60**	0.00	0.00	0.00	0.00	0.00
(*A veces me gusta cotillear sobre los demás*)						
2. I find it very difficult to show my feelings	-0.01	0.12	**0.76**	-0.03	0.06	-0.09
(*Me cuesta mucho mostrar mis sentimientos*)						
6. I keep my feelings to myself	-0.06	0.18	**0.73**	0.03	0.07	-0.13
(*Me guardo mis sentimientos para mí mismo/a*)						
10. I like to show what I am feeling	-0.05	0.10	**-0.74**	-0.01	-0.07	0.06
(*Me gusta demostrar lo que siento*)						
14. I hide my emotions	-0.06	0.19	**0.80**	-0.01	0.06	-0.07
(*Escondo mis emociones*)						
19. Other people think I am cold and distant	-0.01	0.02	**0.47**	0.12	0.02	0.03
(*A los demás les parezco frío y distante*)						
23. Other people can immediately tell how I am feeling	0.07	0.37	**-0.71**	0.06	0.08	-0.14
(*Los demás notan en seguida cómo me siento*)						
28. Other people say that I am very expressive	0.18	0.19	**-0.54**	-0.08	0.04	0.06
(*Los demás me dicen que soy muy expresivo*)						
32. People can tell how I am feeling just by looking at me	0.14	0.40	**-0.65**	0.03	0.16	-0.21
(*Se me nota en la cara como me siento*)						
37. Other people can tell at once if I am sad or angry	0.18	0.32	**-0.67**	-0.01	0.04	-0.15
(*Los demás notan enseguida si estoy triste o enfadado*)						
41. I am reserved about my feelings	-0.02	0.17	**0.77**	-0.04	0.00	-0.07
(*Soy reservado/a respecto a mis sentimientos*)						
3. I do what I like even if it might be detrimental to other people	0.27	0.11	0.06	**0.43**	0.03	0.13
(*Hago lo que quiero, aunque perjudique a los demás*)						
7. I take advantage of others	0.23	0.10	0.00	**0.68**	-0.03	0.05
(*Me aprovecho de los demás*)						
11. I feel bad when I hurt others	-0.13	0.36	-0.01	**-0.60**	0.03	0.04
(*Me siento mal cuando perjudico a otras personas*)						
15. I feel bad when I hurt someone	-0.17	0.37	0.07	**-0.63**	0.07	-0.07
(*Me siento mal cuando hago daño a alguien*)						
20. Seeing other people’s misfortunes upsets me	-0.03	0.37	-0.03	**-0.63**	-0.08	0.11
(*Me entristece ver las desgracias de la gente*)						
24. I seldom apologise when I make a mistake	-0.02	0.11	0.00	**0.29**	0.07	-0.08
(*Raramente pido perdón cuando me equivoco*)						
29. I care about others	0.00	0.30	-0.10	**-0.66**	-0.07	0.05
(*Me preocupo por los demás*)						
34. It is logical that clumsy people are made fun of	0.22	-0.07	-0.07	**0.40**	0.05	0.06
(*Es lógico que la gente se burle de las personas torpes*)						
38. I feel bad for people who are worse off than I am	-0.08	0.34	-0.05	**-0.60**	-0.01	0.08
(*Me siento mal por las personas que están peor que yo*)						
40. I am often very pleasant with people I do not like so that	0.16	0.04	-0.03	**0.45**	-0.12	0.07
I can get something out of them						
(*A menudo soy muy agradable con personas que me caen mal, para conseguir algo de ellas*)						
43. I sometimes use others to get what I want	**0.41**	0.10	-0.04	**0.63**	-0.05	0.09
(*A veces utilizo a los demás para conseguir lo que quiero*)						
4. I always do things at the last minute	**0.30**	0.08	0.00	-0.09	**0.67**	0.04
(*Siempre hago las cosas en el último momento*)						
8. I try to do my work as well as I can	-0.17	0.26	-0.04	-0.02	**-0.62**	-0.07
(*Me preocupo por hacer mis tareas de la mejor forma posible*)						
12. I generally finish what I start	-0.25	0.25	0.04	0.05	**-0.61**	0.03
(*Generalmente acabo lo que empiezo*)						
16. I avoid my responsibilities	0.18	0.00	0.07	0.06	**0.45**	0.05
(*Evito las responsabilidades*)						
21. I fall behind with my obligations	0.24	0.09	0.04	-0.07	**0.73**	0.03
(*Me retraso en el cumplimiento de mis obligaciones*)						
25. I like order	-0.09	0.26	-0.01	-0.01	**-0.41**	-0.06
(*Me gusta el orden*)						
30. I prefer to get my work done quickly, even if the result is	0.26	0.05	-0.04	0.09	**0.55**	0.03
not as good as it could be						
(*Prefiero acabar mis tareas rápidamente, aunque el*						
*resultado sea peor*)						
35. I invest time and trouble on my studies and my work	-0.15	0.19	0.01	-0.06	**-0.59**	-0.16
(*Invierto tiempo y esfuerzo en los estudios o en el trabajo*)						
39. I try not to waste time	-0.06	0.26	0.05	-0.02	**-0.49**	0.13
(*Evito perder el tiempo*)						
5. I do not like the idea of taking drugs	-0.10	0.23	0.01	0.03	-0.04	**-0.33**
(*Me disgusta la idea de consumir drogas*)						
9. I often enjoy doing illegal things	0.21	0.08	0.00	0.02	-0.05	**0.80**
(*A menudo me divierto haciendo cosas ilegales*)						
13. I try to follow the rules	-0.18	0.24	-0.03	-0.05	-0.17	**-0.58**
(*Intento seguir las reglas*)						
17. I am a rebel	0.16	0.07	-0.05	0.06	0.11	**0.60**
(*Soy una persona rebelde*)						
22. I have occasionally had legal problems	-0.02	0.09	-0.05	0.15	0.12	**0.53**
(*En alguna ocasión he tenido problemas con la ley*)						
26. I have a great deal of respect for authority	0.00	0.22	-0.02	0.02	-0.02	**-0.72**
(*Siento mucho respeto por la autoridad*)						
31. I believe that the law must be respected	-0.07	0.23	-0.03	0.05	0.01	**-0.79**
(*Creo que es necesario respetar las leyes*)						
36. It is fun to graffiti on walls	0.17	0.01	0.03	0.00	0.00	**0.63**
(*Es divertido hacer pintadas en las paredes*)						
Factor reliability	0.90	0.89	0.96	0.91	0.93	0.92
Congruence index			0.98	0.96	0.96	0.91


*In bold, expected salient loadings. SD, social desirability; AC, acquiescence; UE, unemotional; CA, callousness; UC, uncaring; AB, antisocial behavior.*

**Table 2:** Interfactor correlation matrix.

Measure	UE	CA	UC	AB
UE	–			
CA	0.049	–		
UC	0.044	0.062	–	
AB	0.024	0.230	0.248	–


*UE, unemotional; CA, callousness; UC, uncaring; AB, antisocial behavior.*

### Item Analyses

We computed the descriptive statistics and discrimination indices for the 42 items. The means ranged between 1.51 and 4.26, and the standard deviations between 0.78 and 2.47. Discrimination indices reached values higher than 0.14 with a maximum of 0.65, which indicates that the items of each INCA subscale are correlated with each other.

### Scale Analyses

Likewise, we computed the reliability estimates on the basis of the factor scores for each scale (see, e.g., Mellenbergh, 1994). As can be seen in the penultimate line of Table 1, the reliabilities for the content subscales ranged between ρ_θθ_ = 0.90 and ρ_θθ_ = 0.96, which indicates a good reliability for the factor scores.

Regarding the convergent and discriminant validity of INCA, Table 3 shows the correlations between the INCA factor score estimates and the ICU, BIS-11c, and OPERAS questionnaires. It should be taken into account that, according to Cohen (1988), correlations of 0.50 or higher have a large effect size, correlations around 0.30 have a medium effect size and correlations around 0.10 have a small effect size. As can be seen, the unemotional, callousness and uncaring scores of INCA have the highest correlation with their corresponding ICU scales. The antisocial behavior subscale presents moderate correlations with INCA’s callousness and uncaring scores. Moreover, as expected, there are significant correlations between the INCA scores and impulsivity. More specifically, the INCA factor scores that are most related to impulsivity are uncaring and antisocial behavior, which are positively correlated with motor and non-planning impulsivity of BIS-11c, while uncaring is negatively correlated with cognitive impulsivity. In addition, callousness has a positive although small correlation with motor impulsivity. There are also significant correlations with some personality traits of the test OPERAS. More specifically, callousness and antisocial behavior factor scores are negatively correlated with the traits conscientiousness and agreeableness. Callousness also has a significant negative correlation with openness to experience.

**Table 3:** Pearson correlation coefficients and structural validity coefficients between INCA, ICU, BIS-11c, and OPERAS.

		INCA			
		
		UE	CA	UC	AB
ICU	UE	0.76^∗∗^	0.09	0.04	-0.04
		(0.87)	(0.11)	(0.05)	(-0.05)
	CA	0.22^∗∗^	0.47^∗∗^	0.22^∗∗^	0.55^∗∗^
		(0.26)	(0.56)	(0.26)	(0.66)
	UC	-0.04	0.17^∗^	0.43^∗∗^	0.45^∗∗^
		(-0.05)	(0.20)	(0.49)	(0.52)
BIS-11c	MI	-0.08	0.20^∗^	0.41^∗∗^	0.49^∗∗^
		(-0.09)	(0.23)	(0.48)	(0.57)
	N-PI	0.09	0.15	0.52^∗∗^	0.39^∗∗^
		(0.11)	(0.18)	(0.63)	(0.48)
	CI	-0.09	0.02	-0.36^∗∗^	-0.12
		(-0.11)	(0.03)	(-0.45)	(-0.15)
OPERAS	EX	-0.41^∗∗^	-0.07	-0.12	0.13
		(-0.45)	(-0.08)	(-0.13)	(0.15)
	ES	-0.11	-0.04	-0.17^∗^	-0.13
		(-0.12)	(-0.05)	(-0.19)	(-0.15)
	CO	-0.14	-0.18^∗^	-0.63^∗∗^	-0.38^∗∗^
		(-0.16)	(-0.21)	(-0.74)	(-0.45)
	AG	-0.07	-0.35^∗∗^	-0.07	-0.24^∗∗^
		(-0.08)	(-0.43)	(-0.09)	(-0.30)
	OE	-0.15	-0.26^∗∗^	-0.21^∗∗^	0.01
		(-0.17)	(-0.30)	(-0.24)	(0.01)


*The figures in parentheses are the structural validity coefficients. UE, unemotional; CA, callousness; UC, uncaring; AB, antisocial behavior; MI, motor impulsivity; N-PI, non-planning impulsivity; CI, cognitive impulsivity; EX, extraversion; EE, emotional stability; CO, conscientiousness; AG, agreeableness; OE, openness to experience. ^∗∗^*p* < 0.01, ^∗^*p* < 0.05.*

Uncaring scores have a negative correlation with emotional stability and openness to experience, but these correlations have a small effect size because they are lower than 0.30. Uncaring also has a negative correlation with conscientiousness, and a large effect size because this correlation is higher than 0.50. Finally, the unemotional factor scores have a negative correlation only with extraversion, and a medium effect size because this correlation is lower than 0.50.

**FIGURE 1:**
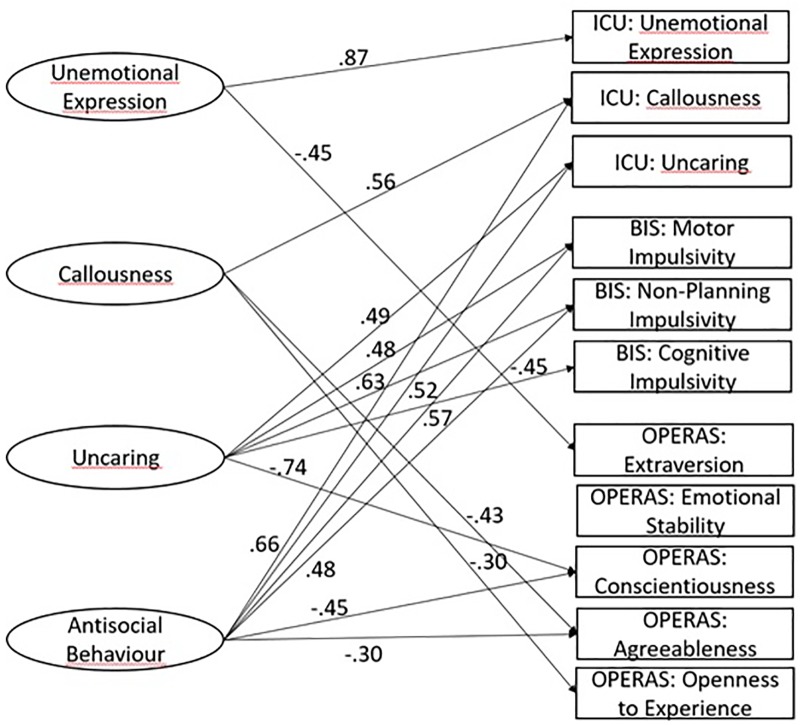
Structural part of the model with the substantial relations between the factors and the external variables from ICU, BIS-1lc, and OPERAS.

In order to improve the raw validity estimates described above, we also extended the measurement model into a full structural model that includes the relations between the factors and the relevant external variables (the subscales of the questionnaires ICU, BIS-11c, and OPERAS). Given the complexity and size of the full model, however, we decided to include these additional variables as single indicators. In this setting, and provided that the unique parts of the original items are uncorrelated with the additional variables (which is expected because they do not share specific content), the relative fit of the full structural model is expected to be the same as that of the measurement model, and that is the result we obtained. However, the validity path estimates can now be interpreted as disattenuated correlations in which the measurement errors of both the factor score estimates and the relevant related variables have been corrected. Therefore, Table 3 provides both the uncorrected validity coefficients and the structural validity coefficients (free from measurement error). Moreover, Figure 1 shows the structural part of the model in which the latent variables or factors are related to the external variables derived from ICU, BIS-11c, and OPERAS. However, for the sake of clarity, only the weights that can be considered as substantial are shown in the figure. Otherwise, the graph of the structural sub-model would be too complex to be clearly interpreted. Given that the structural weights in this case can be essentially interpreted as product-moment correlations, we have considered the 0.30 to be a reasonable threshold for considering a weight to be substantial. This choice stems from the fact that the effect size for a correlation coefficient is the correlation itself, and, according to Cohen (1988), effect sizes below 0.30 should be considered as small.

Table 4 shows the means of the factor scores (on a T scale metric) in boys and girls. Because the individual measurement errors of the factor score estimates are expected to cancel, the difference tests on Table 4 can be regarded as structural comparisons based on latent means. It should be pointed out that the direct structural estimation of the latent mean differences would have been a theoretically superior to the procedure that we used here. However, this approach would have involved fitting a very complex multiple-group model likely to lead to unstable results. So, we believe that our chosen approach is a good trade-off that, as discussed above, is expected to provide consistent results. As can be seen, there were significant sex differences only for callousness, with lower scores for women [*t*_(716)_= -5.46; *p* < 0.01] and a medium effect size (*d* = 0.41).

**Table 4:** Mean scores for men and women on INCA scales and effect sizes.

	Men Mean (SD)	Women Mean (SD)	*t*	*p*	*d*
UE	50.47 (9.45)	50.12 (11.10)	*t*_(712)_ = -0.46	0.65	–
CA	52.34 (10.42)	47.96 (10.86)	*t*_(716)_ = -5.46	<0.01	0.41
UC	50.70 (10.26)	49.55 (10.77)	*t*_(716)_ = -1.44	0.15	–
AB	50.37 (11.49)	48.78 (11.16)	*t*_(716)_ = -1.87	0.06	–


*UE, unemotional; CA, callousness; UC, uncaring; AB, antisocial behavior.*

## Discussion

The results presented above indicate that the INCA has suitable psychometric properties, good reliability, the expected factor structure and good validity coefficients. The traditional CU scales measured by the INCA are similar to those proposed by the ICU, but their content shows a clear and replicable factor structure, as is shown by the fact that the same factor structure has been found in two different subsamples. Furthermore, all scales provide scores that are free of SD and AC response biases. As we mentioned in the introduction, this kind of scale is often affected by SD, and the loadings of some items on SD justified controlling this bias. On the other hand, several studies have shown that controlling AC makes the factor structure simpler and more congruent (Rammstedt and Farmer, 2013; Navarro-González et al., 2016; Morales-Vives et al., 2017), which is the case of INCA. In addition, since these results were obtained when the test was administered anonymously, we assume that when the test is not anonymous, such as in forensics (penitentiary centers, social services, etc.), adolescents would show a greater SD and hide their psychopathic traits even more. Therefore, it would be particularly advisable to control response biases in these situations.

INCA also has good convergent and discriminant validity. In fact, the test’s subscales had the expected relationships with the measures of several constructs traditionally linked with psychopathy, such as impulsivity and agreeableness. Our findings support the associations between high levels of CU traits and impulsivity found in previous studies (Roose et al., 2010; López-Romero et al., 2015). These associations might be explained by the lack of inhibition common in the aggressive and impulsive behaviors that psychopaths usually display. The criteria validity of the INCA was also supported by its relationships with the Big Five personality traits. As previous studies have pointed out, conscientiousness and agreeableness were the dimensions that were most related to CU traits (Miller and Lynam, 2001; Lynam et al., 2005; Essau et al., 2006). More specifically, there was a negative correlation between agreeableness and CA and between conscientiousness and UC. The negative relationship between conscientiousness and UC was expected, because UC involves a lack of responsibility and effort, which implies a low level of conscientiousness. Therefore, young people with higher UC traits tend to show irresponsible behavior characterized by lack of perseverance, disorganization and poor work orientation. Likewise, we also expected to find a negative relationship between agreeableness and CA, because both dimensions share empathy as the key feature. This implies that adolescents with higher levels of CA are less nice and considerate with others. Another noteworthy result is the negative relationship between the OE scale and the UC traits, specifically CA and UC. These findings are similar to those of Duran-Bonavila et al. (2017), in which juvenile delinquents and young people at risk of social exclusion scored lower than the community sample on OE, probably because this personality trait is related to intellectual curiosity and cultural interests which may be less stimulated in the marginal environments of society.

Previous studies have obtained contradictory results in terms of sex differences in CU traits. In fact, while some studies found higher scores in boys for CA, UC, and UE traits (Essau et al., 2006; Fanti et al., 2009), others only found higher scores in boys for UC and UE traits (Ciucci et al., 2014). Houghton et al. (2013) did not report significant differences between boys and girls in a sample of community children. We only found significant sex differences in the CA subscale, with higher scores in boys, which is congruent with the studies by Essau et al. (2006) and Fanti et al. (2009). This may be because CA is strongly associated with low AG (Miller and Lynam, 2001; Lynam et al., 2005; Essau et al., 2006), a dimension in which women tend to score higher than men (Schmitt et al., 2008). Further studies are needed to elucidate which of the CU traits are most affected by sex differences.

It should be mentioned that the INCA scores have to be interpreted as factor scores, not as raw additions of individuals’ answers to the items (raw scores). To compute factor scores, the individuals’ answers to items must be standardized by using the means and standard deviations of the items, and the standardized responses must be added as a weighted addition. Finally, the total addition should be transformed from typical scores to T scores (i.e., mean 50 and standard deviation 10). In addition, normalized percentiles should be computed as part of a proper psychological report. Although this procedure is not complex, it is not yet straightforward for applied psychologists. To solve this drawback, we have developed a public internet application that applied psychologists can use to obtain factor scores and normalize percentiles. It is free share software available at http://psico.fcep.urv.cat/links/INCAS_CORRECTOR.zip.

This study has some limitations that should be addressed in future research. In fact, it is based only on a community sample, so future studies should analyze the results in samples with high levels of antisocial behavior, such as juvenile offenders. This kind of sample would also be particularly useful in providing information about the predictive validity of the INCA to identify young people who might have severe behavioral and psychosocial maladjustment. Moreover, further studies should be carried out in order to assess the correlations between some specific facets of the Big Five, such as warmth, positive emotions, assertiveness or excitement, and the dimensions of the test.

Despite these limitations, the study provides evidence about the psychometric properties of INCA, and shows that this instrument is a useful measure of CU traits in adolescents. In this regard, INCA might be especially valuable in assessment as well as in the prevention and intervention programs of some applied fields such as forensics, education and mental health.

## Author Contributions

FM-V contributed to the design of the study and collection of data, carried out most of the statistical analyses, supervised the research and revised it critically, wrote part of the article, and provided the final approval of the version to be published. SC contributed to the collection of data and contacted with some centers and wrote part of the article. UL-S contributed to the design of the study, carried out part of the statistical analyses, and revised the research critically. AV-C formulated the research question, was responsible for the statistical design of the study, supervised the research and revising it critically, and provided the final approval of the version to be published.

## Conflict of Interest Statement

The authors declare that the research was conducted in the absence of any commercial or financial relationships that could be construed as a potential conflict of interest.
